# Machine Learning Models for Glycemic Prediction in Type 1 and Type 2 Diabetes: Systematic Review and Meta-Analysis

**DOI:** 10.2196/100653

**Published:** 2026-11-09

**Authors:** Haoran Yang, Diliyaer Abudukeremu, Yuhan Shi, Li Fang, Lei Li, Wenzheng Gu, Yunqi Jia, Rongfang He

**Affiliations:** 1 Department of Pathology, The First Affiliated Hospital, Hengyang Medical School, University of South China, No. 69, Chuanshan Road, Hengyang, 421001, China, +86 18773464745; 2 Tianjin Medical University Cancer Institute and Hospital, Tianjin, China; 3 The Second Clinical College of Tianjin Medical University Second Hospital, Tianjin, China; 4 Meiwan Community Health Service Station (Municipal Party Committee Clinic), Yongzhou, China; 5 Cai Township Health Center, Yongzhou, China; 6 First Teaching Hospital of Tianjin University of Traditional Chinese Medicine, National Clinical Research Center for Chinese Medicine, Tianjin, China

**Keywords:** diabetes mellitus, type 1 diabetes, type 2 diabetes, continuous glucose monitoring, blood glucose forecasting, hypoglycemia, hyperglycemia, machine learning, artificial intelligence, systematic review, meta-analysis

## Abstract

**Background:**

Machine learning models are increasingly used for glycemic event prediction and continuous glucose forecasting, but targets, populations, horizons, validation, and uncertainty reporting remain heterogeneous.

**Objective:**

This study aimed to evaluate machine learning models for glycemic prediction in type 1, type 2, and mixed diabetes populations while distinguishing the full evidence map from the quantitatively analyzable subset.

**Methods:**

We searched 9 English- and Chinese-language databases from inception to April 12, 2026, for studies predicting future glycemic events or glucose values in people with diabetes. Continuous forecasting and binary event prediction were analyzed separately. Root-mean-square error (RMSE) was the principal continuous end point; mean absolute error was supportive. Area under the receiver operating characteristic curve was synthesized using a Bayesian random-effects model because analyzable 2×2 data were sparse. Two reviewers (HY and DA) independently applied PROBAST (Prediction Model Risk of Bias Assessment Tool) to 19 quantitative-source reports (28 assessment units).

**Results:**

We identified 787 records, assessed 310 full texts, and included 299 reports representing 270 studies; 19 reports contributed to quantitative synthesis. The review included 128 type 1, 26 type 2, 78 mixed, and 38 unclear-diabetes-type studies. Pooled RMSE was 1.18 mmol/L (95% CI 1.01‐1.34; 37 effects), supportive mean absolute error was 0.90 mmol/L (95% CI 0.78‐1.02; 20 effects), and Bayesian pooled area under the receiver operating characteristic curve was 0.926 (95% credible interval 0.877‐0.957). Supportive sensitivity and specificity were 0.77 and 0.80. Of 28 PROBAST units, 24 were high risk, 2 unclear, and 2 low. Post hoc exploratory within-report analysis estimated an RMSE increase of 0.481 mmol/L per additional 30 minutes (95% CI 0.213‐0.749), but overlap sensitivity reduced certainty. In the 270-study clinical readiness audit, external validation occurred in 4.0% (10/251), prospective evaluation in 0.7% (2/270), calibration in 4.8% (13/270), decision-curve or net-benefit analysis in 2.6% (7/270), and implementation or human-factors evaluation in 2.6% (7/270).

**Conclusions:**

Glycemic prediction studies show forecasting and discrimination signals, but heterogeneity, high risk of bias, incomplete uncertainty reporting, and limited external and prospective validation preclude deployable benchmarks. Pooled estimates should be viewed as summaries of analyzable literature. Future studies need transparent horizon, threshold, calibration, validation, uncertainty, and implementation reporting.

## Introduction

### Clinical and Digital Health Context

Diabetes involves both sustained hyperglycemia and short-term glucose variability, with glycemic excursions affecting safety and quality of life. Continuous glucose monitoring (CGM), connected insulin delivery, meal logs, and wearable sensors now provide high-frequency data for studying and predicting glucose dynamics.

Machine learning methods can model nonlinear, multimodal glucose data, but studies differ in diabetes phenotype, target, inputs, forecast horizon, validation, dataset provenance, and reporting. Binary glycemic event prediction and continuous glucose forecasting are often discussed together despite requiring different outcomes, effect measures, and clinical interpretations.

The central challenge is evidence transparency and transportability rather than model availability. Many reports omit uncertainty, reuse datasets, or provide results unsuitable for synthesis; we therefore distinguish the broad systematic review evidence map from the smaller quantitative subset. Clinical use also differs by diabetes type. Type 1 diabetes studies often address insulin therapy, hypoglycemia prevention, sensors, and decision support [1-5], whereas type 2 diabetes studies include dietary personalization, inpatient management, medication effects, and broader excursions [6,7].

### Rationale for Separating Prediction Tasks

Binary event prediction asks whether hypoglycemia or hyperglycemia will occur within a specified horizon, whereas continuous forecasting estimates a future glucose value or trajectory. Their effect measures and implications for action are not interchangeable. Accordingly, the broad review maps the field, while formal synthesis is limited to reports with usable uncertainty or uniquely reconstructable threshold information. We did not present a single pooled benchmark as directly deployable across populations, treatments, and horizons.

Root-mean-square error (RMSE) and mean absolute error (MAE) summarize continuous forecast error, whereas area under the receiver operating characteristic curve (AUROC), sensitivity, and specificity describe binary discrimination or threshold classification. These measures support different clinical uses and were analyzed separately [1,5,7,8]. This prediction-family framework links each estimate to the evidence that can support it and identifies where only descriptive synthesis is justified.

### Objectives

We aimed to (1) systematically review machine learning studies of glycemic event prediction and continuous glucose forecasting in type 1, type 2, or mixed diabetes populations; (2) synthesize reports with sufficient uncertainty or uniquely reconstructable threshold data; (3) examine heterogeneity related to horizon, validation, publication status, and dataset provenance; and (4) interpret transportability. We also audited all 270 studies against 8 predefined clinical readiness criteria.

## Methods

### Design, Registration, and Reporting

We conducted a systematic review and meta-analysis following PRISMA (Preferred Reporting Items for Systematic Reviews and Meta-Analyses) 2020 and, for threshold syntheses, PRISMA-DTA (Preferred Reporting Items for Systematic Reviews and Meta-analyses of Diagnostic Test Accuracy Studies) [9,10]. PROBAST (Prediction Model Risk of Bias Assessment Tool) [11] was applied to the 19 reports contributing to quantitative synthesis; the broader 270-study map was characterized by reporting and validation features without formal PROBAST judgments. TRIPOD (Transparent Reporting of a Multivariable Prediction Model for Individual Prognosis or Diagnosis) concepts informed interpretation [12].

The protocol was finalized in early April 2026, and PROSPERO registration (CRD420261366978) was completed on April 12, 2026. The search was frozen that day; reconciled full-text assessment, extraction, and analysis followed. Before definitive fitting, Bayesian hierarchical AUROC was selected as the principal binary synthesis because analyzable 2×2 data were sparse, and RMSE was prioritized over MAE because variance-ready RMSE evidence was stronger. The review is therefore protocol-informed with documented presynthesis refinements rather than fully day 0 preregistered.

Continuous forecasting and binary event prediction remained separate throughout, as did review-level and meta-analysis–level evidence. Final updating refreshed searches, harmonized evidence tables, removed nonextractable abstract- or proceedings-only records, and added diabetes-type and horizon analyses. Full peer-reviewed conference papers were retained only when usable full text met the same criteria as journal papers.

### Information Sources and Search Strategy

We searched PubMed, Web of Science Core Collection, Embase, Scopus, IEEE Xplore, CNKI, ACM Digital Library, arXiv, and the Cochrane Library from database inception to April 12, 2026. Search terms combined diabetes-related terms, glycemia or CGM terms, machine learning or AI terms, and prediction or forecasting terms. The complete source-specific search strategies and search dates are provided in Table S1 in Multimedia Appendix 1.

The auditable supplementary package is distributed across Multimedia Appendix 2 [13-16], Multimedia Appendix 3 [7,13-28], Multimedia Appendix 4 [1,2,7,13-21,23,25,26,28-47], and Multimedia Appendix 5. Multimedia Appendix 2 contains the search, screening, study characteristics, historical extraction, derivation, and figure materials; Multimedia Appendices 3–5 contain the PROBAST audit, source-verified analysis tables, and complete dual-review clinical readiness records.

No language restriction was applied at search. The final evidence base comprised 266 English- and 4 Chinese-language studies. Bilingual reviewers extracted Chinese reports; translated text was used only for verification. No otherwise eligible report in another language required quantitative extraction. Preprints and engineering reports had to meet the same human-diabetes, future-prediction, and extractable performance criteria as journal papers. Studies published after April 12, 2026, and records without usable full text were excluded.

### Eligibility Criteria

Eligible reports were original empirical studies of machine learning or AI for predicting future glycemic events or glucose values in type 1, type 2, mixed diabetes populations, or validated diabetes glucose dynamics datasets. We excluded diagnostic or complication classification, treatment recommendation without a future glycemic end point, healthy or prediabetes-only populations, nonhuman or in vitro studies, reviews, patents, theses, abstracts, and nonextractable proceedings. Full peer-reviewed conference papers were eligible when their full text supported the same screening and extraction process.

Quantitative synthesis required additional data readiness. Continuous outcomes needed MAE or RMSE with usable or defensibly derivable uncertainty; AUROC needed a SE, CI, or defensible derivation; and threshold outcomes needed reported or uniquely reconstructable true positive (TP), false positive (FP), false negative (FN), and true negative (TN). Other eligible studies remained in the narrative evidence base. At full text, we excluded reports without a future glucose value or event target, a human diabetes population, usable full text, reconcilable population eligibility, or eligible performance data. Each excluded report received 1 standardized primary reason.

### Selection Process and Data Extraction

Two reviewers (HY and DA) independently screened records, assessed full texts, and extracted data; disagreements were resolved by discussion or senior review (YJ and WG). Variables included study and population characteristics, dataset provenance, target, threshold, inputs, horizon, model family, validation, calibration, and performance metrics. Diabetes type was coded as type 1, type 2, mixed, or unclear using the report and dataset description. Public datasets with a documented diabetes type retained that classification; inseparable mixed populations were not reassigned.

Input modality reflected model inputs: CGM-only models were separated from models incorporating insulin, meals, activity, demographics, laboratory values, or other context. Horizons were recorded in minutes, with multiple eligible horizons retained as separate effects under a shared study-cluster identifier. Performance data were extracted preferentially from tables and then text, supplements, or clearly scaled figures. Each derived value, unit conversion, and uncertainty calculation were logged for audit. Multiple eligible models, horizons, thresholds, or validation strata were stored as separate estimates sharing a study-cluster identifier. Original and converted values, uncertainty source, derivation, diabetes type, horizon, input modality, and analysis tier were retained.

### Source-Fidelity and Dataset-Overlap Audit

Before conducting the additional revision-stage analyses, we rechecked all 80 effect rows from the 19 quantitative-source reports against the full text. For each row, the audit retained the source-reported metric and unit, analytic population or denominator, forecast horizon, validation context, full-text page location, and a supporting excerpt. Corrections were made only when the article-level evidence showed that a value, unit, population, or uncertainty field had been carried forward incorrectly. RMSE values were not treated as SDs, threshold tables were retained only when the 2×2 cells were directly reported or uniquely reconstructable, and simulation-only evidence was excluded from the primary clinical synthesis. The final locked quantitative-source set included Zhang et al [16] and excluded Mohebbi et al [48].

Dataset and cohort provenance was audited at report level. Reports were linked when the full text identified the same named public dataset, participant cohort, institution and recruitment period, or an otherwise traceable shared data source. Five reports using the OhioT1DM benchmark family were flagged as a confirmed overlap family. Dataset-family identifiers were retained in the analysis files so that overlap could be addressed by conservative clustering and exclusion sensitivity analyses rather than by silently treating every report as independent.

Values read from figures were used only when the axis scale, plotted point, and uncertainty limits were visually distinguishable. These values were flagged as figure-derived and were not prioritized over tabulated or text-reported values. If the figure did not permit a reliable reading of either the point estimate or uncertainty interval, the study was retained in the systematic review evidence table but not used for inverse-variance pooling.

### Outcome Families and Metric Handling

Binary event prediction and continuous forecasting were analyzed separately. MAE and RMSE were converted using 1 mmol/L=18 mg/dL. RMSE was the principal continuous end point because it had the strongest variance-ready evidence; MAE was supportive and analyzed separately. Continuous-outcome uncertainty was standardized as an SE in mmol/L. From a 95% CI, SE=(upper-lower)/(2×1.96); from an SD and independent analytic sample size, SE=SD/square root(n). Estimates and uncertainty in mg/dL were divided by 18. Derivations were used only when the source metric, denominator, and validation set were clear.

Reported AUROC SEs were used directly. From a coherent 95% CI within 0‐1, uncertainty was derived on the logit(AUROC) scale as [logit(upper)-logit(lower)]/(2×1.96). Probability-scale SEs were transformed by the delta method. AUROC estimates without usable uncertainty were excluded from the principal model. For threshold prediction, reported 2×2 tables were preferred. Unique reconstructions used reported sensitivity and specificity with fixed event and nonevent denominators; rounded TP, FP, FN, and TN had to reproduce the reported totals and metrics. We rejected nonunique reconstructions and never assumed an arbitrary prevalence or sample split.

Continuous uncertainty came from compatible SEs, CIs, prediction-error SDs, or other clearly mapped summaries. We did not impute SDs solely to enlarge the main analysis; indirect derivations were restricted to expanded or maximal sensitivity datasets. RMSE and MAE remained separate because they weight forecast error differently. Accuracy, *F*_1_-score, precision, recall, and other classification metrics were extracted when available for descriptive purposes, but they were not used as primary pooled outcomes unless they could be mapped to AUROC or a threshold-based 2×2 structure. This decision avoided mixing metrics that depend on threshold, prevalence, and class balance in different ways.

### Study Clusters, Dataset Reuse, and Validation

A study cluster was defined as a citation-level or dataset-level grouping within which multiple models, horizons, targets, or validation splits could produce several correlated effect estimates. This term was used because a single paper could contribute more than 1 performance estimate and because some public datasets were reused across multiple publications. Cluster identifiers were used to avoid treating all extracted effects as fully independent. Validation design was classified as internal random or cross-validation, held-out same-cohort testing, temporal testing, external cohort or external institutional validation, or unclear. These features informed both study characterization and the prespecified PROBAST operational rules for participant-level separation, overlapping windows, future-information leakage, preprocessing and imputation, tuning, test-set reuse, repeated measurements, predictor availability, and calibration.

A study cluster could therefore correspond to a single paper, a repeated public dataset, or a closely related set of effects from the same cohort. The term does not imply that each cluster was a multicenter study. It is an analytic device for handling dependence among multiple extracted estimates. This definition was used consistently in extraction tables, sensitivity analyses, and figure legends. Dataset reuse was especially relevant for public CGM datasets. Multiple models can be evaluated on the same benchmark, sometimes with different preprocessing, horizon definitions, or validation splits. Treating every such estimate as independent would overstate precision. The cluster-aware and leave-one-cluster-out analyses were designed to test whether the main findings depended on a small number of repeated data sources.

### Risk of Bias and Applicability Assessment

Two reviewers (HY and DA) independently assessed the 19 reports contributing to at least 1 quantitative synthesis using the standard PROBAST framework. Assessments were conducted at the prediction model or model group–prediction target–validation population level. Reports containing development and external validation analyses, distinct populations, simulated and clinical datasets, or prediction targets that could yield different domain judgments were divided into separate assessment units. Models were grouped only when they shared participants, predictors, outcome definition, and analytical and validation procedures such that their PROBAST judgments could not reasonably differ. This process yielded 28 assessment units from 19 reports; it did not create 28 independent studies.

Each reviewer independently answered the 20 signaling questions using yes, probably yes, probably no, no, no information, or not applicable, and then assigned judgments for the Participants, Predictors, Outcome, and Analysis domains. Applicability was assessed for Participants, Predictors, and Outcome. The operational rules were prespecified interpretations of standard PROBAST for machine learning and continuous glucose-monitoring studies and not a separate scoring system. No numeric quality score or quality weighting was used.

Disagreements were resolved by consensus after reexamination of the cited full-text evidence and the prespecified rules, with consultation of YJ or WG available when required. Overall risk of bias was low only when all 4 domains were low, high when at least 1 domain was high, and unclear otherwise. Agreement was summarized using observed agreement, unweighted Cohen κ, and Gwet AC1 (agreement coefficient 1). Because questions and assessment units were nested within source reports, 95% CIs were obtained with 2000 study-cluster bootstrap resamples at the report identifier (Study_ID) level. Detailed unit definitions, signaling judgments, consensus rationales, and final assessments are provided in Multimedia Appendix 3.

### Statistical Analysis

Continuous outcomes were synthesized with inverse-variance random-effects models in R (R Foundation for Statistical Computing) using established meta-analysis workflows [49-51]. The principal RMSE analysis used the conservative main dataset. Expanded and maximal datasets were used as sensitivity or exploratory analyses when uncertainty had to be derived more indirectly. Exploratory subgroup analyses examined diabetes type, forecast horizon, and input modality. Horizon categories were ≤15 minutes, 16‐30 minutes, 31‐45 minutes, 46‐60 minutes, and >60 minutes. Because horizons longer than 60 minutes were sparse and clinically different from short-term alerting, they were reported separately and not used to support strong clinical claims.

Binary AUROC was synthesized using a Bayesian normal-normal random-effects model on the logit(AUROC) scale. This was retained as the primary binary synthesis because directly analyzable 2×2 data were insufficient for a stable primary threshold-based meta-analysis. A frequentist restricted maximum likelihood (REML) model on the same scale was used as a sensitivity analysis. Threshold-based sensitivity and specificity were summarized as supportive analyses using logit-transformed random-effects models. Full analysis scripts, input data, derived uncertainty fields, and figure-generation outputs are archived with the revision package.

For clinical translation, we used the supportive pooled sensitivity and specificity to estimate positive and negative posttest probabilities at illustrative pretest probabilities of 5%, 15%, and 30%. These pretest probabilities were review-defined low-, moderate-, and higher-risk scenarios rather than accepted disease-domain standards. They were chosen to show how the same model performance may translate differently across clinical contexts.

Clinical translation calculations used standard likelihood-ratio formulas. The positive likelihood ratio was sensitivity/(1−specificity), and the negative likelihood ratio was (1−sensitivity)/specificity. Pretest probability was converted to pretest odds as p/(1−p). Posttest odds were calculated as pretest odds × likelihood ratio, and posttest probability was then calculated as posttest odds/(1+posttest odds). These values were used for interpretation only and were not treated as a decision threshold for clinical deployment.

The Bayesian AUROC model was selected for the primary binary synthesis for 2 reasons. First, AUROC was more commonly reported with usable uncertainty than full threshold tables. Second, the Bayesian normal-normal structure allowed uncertainty and between-study heterogeneity to be expressed directly on the logit(AUROC) scale while avoiding unstable pooling of sparse reconstructed 2×2 tables as the main binary result. Posterior summaries were reported as medians with 95% credible intervals. The threshold analyses were still retained because they are more clinically interpretable.

Subgroup analyses were specified as exploratory because they were observational comparisons across studies rather than randomized comparisons within studies. We report the number of model-horizon effect estimates and source titles for each subgroup to make the evidence density transparent. Subgroups with very few titles are described but not used to support strong inferential claims. Reporting-bias analyses were also exploratory. Funnel plots were generated for outcomes with enough effects, but asymmetry tests were not treated as definitive because effects were clustered, SEs were sometimes derived, and clinical and methodological heterogeneity were high.

### Continuous Prediction-Horizon Meta-Regression

As a post hoc exploratory analysis, we evaluated prediction horizon continuously using the 37 source-verified RMSE effects. Effects were first collapsed to 23 report-horizon cells by averaging eligible model estimates within each report and horizon, preventing reports that evaluated many algorithms at the same horizon from receiving disproportionate weight. The primary estimand was the within-report change from that report’s own 30-minute RMSE. Reports therefore had to provide a 30-minute value and at least 1 additional fixed horizon, yielding 12 nonzero horizon contrasts from 8 reports.

The primary model estimated the RMSE change per additional 30 minutes with the intercept constrained to zero at the 30-minute anchor. Equal contrast weights were used because source-reported uncertainty was unavailable for many eligible cells. Dependence was addressed with report-clustered CR2 covariance and Satterthwaite small-sample inference. Sensitivity analyses clustered the confirmed Ohio T1DM reports as 1 dataset family, excluded that family, restricted the data to contrasts with source-reported uncertainty, applied inverse approximate contrast-variance weights, evaluated marginal report-horizon cells, and restored the simulation-only study. Results are reported in both mg/dL and mmol/L, using 1 mmol/L=18.0182 mg/dL.

### Clinical-Readiness Evidence Audit

Across all 270 included studies, we assessed 8 review-defined clinical-readiness criteria: use of real patient data, participant-level or temporal test separation, external validation, prospective evaluation, calibration assessment, threshold-specific operating performance, decision-curve or net-benefit analysis, and implementation or human-factors evaluation. Automated evidence-location prefill was used only to organize candidate evidence and support page-level retrieval; it was not treated as an independent reviewer judgment.

Two reviewers (LF and LL) independently completed all 2160 study criterion judgments (270 studies × 8 criteria). Each reviewer recorded a categorical judgment with supporting evidence and source location. The final consensus for all disagreements and other records requiring explicit resolution was reached jointly by the 2 reviewers or approved by the research team. Agreement was summarized as observed agreement and unweighted Cohen κ. Criterion-specific proportions used Yes/(Yes+No), excluding Unclear and Not applicable judgments from the denominator. The number of criteria met per study was summarized descriptively from final Yes judgments and was not treated as a validated composite score.

## Results

### Study Selection

The updated search identified 787 records from databases and registers (Figure 1). No duplicate records were removed in the final auditable flow because source files were harmonized before final screening. We screened 787 records and excluded 402 at title and abstract levels. We sought 385 reports for retrieval; 75 could not be retrieved. We assessed 310 full-text reports and excluded 11 reports: 8 because the outcome or prediction task was not eligible, 1 because the population was wrong or did not include an eligible human diabetes cohort, and 2 because no extractable eligible clinical performance data were available. The final systematic review set included 299 reports representing 270 studies. Nineteen studies contributed to at least 1 quantitative synthesis (Table 1); these main quantitative source studies are cited in aggregate here [1,2,8,13-16,29-32,34,52-58].

**Figure 1.**
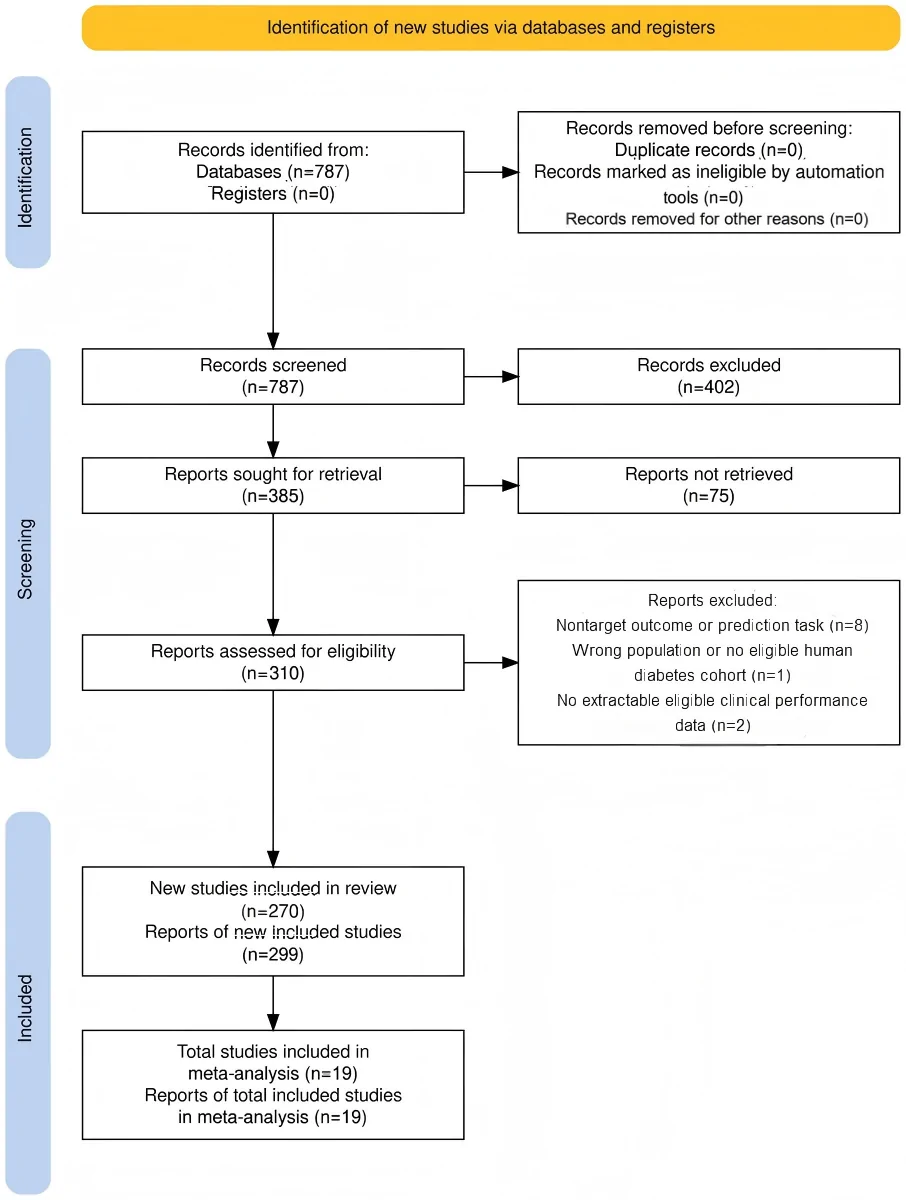
PRISMA (Preferred Reporting Items for Systematic Reviews and Meta-Analyses) 2020 flow diagram.

**Table 1.** Summary of the revised evidence base.

Characteristic	Count	Interpretation
Records identified	787	Records from databases and registers searched to April 12, 2026
Reports assessed for eligibility	310	Full texts assessed after retrieval
Reports included in systematic review	299	Reports representing the final review-level evidence base
Studies included in systematic review	270	Unique included studies after full-text review
Studies included in meta-analysis	19	Studies contributing to at least 1 quantitative synthesis
Type 1 diabetes studies	128	Full review-level count
Type 2 diabetes studies	26	Full review-level count
Studies including mixed populations of type 1 and type 2 diabetes	78	Full review-level count
Unclear diabetes type	38	Full-text reports in which diabetes type could not be confidently classified
Publication year range	2001‐2026	Median publication year 2023; 128 studies published from 2024 onward
Studies with extractable sample size	136	Median 42 participants; IQR 12-206; range 1-37,740
English-language studies	266	Full-text evidence base
Chinese-language studies	4	Extracted by bilingual reviewers

The relatively large gap between the 270 included studies and the 19 studies contributing to meta-analysis reflects reporting limitations rather than lack of relevance. Many included studies reported model rankings, point estimates, or graphical performance summaries but did not provide uncertainty intervals, SEs, SDs, or reconstructable threshold data. These studies informed the narrative synthesis, study-characteristics tables, and methodological interpretation, consistent with guidance for synthesis without meta-analysis when quantitative pooling is not justified [59].

### Characteristics of the Evidence Base

Among the 270 included studies, 128 focused on type 1 diabetes, 26 focused on type 2 diabetes, 78 included mixed type 1 and type 2 diabetes populations, and 38 did not allow confident diabetes-type classification after full-text assessment. Clinical setting was inpatient in 182 studies, outpatient in 44, simulation or simulated-plus-clinical in 7, and unclear in 37. Age group was adult in 72 studies, pediatric in 17, mixed age in 32, and unclear in 149. These distributions show not only that the review-level evidence base is much broader than the meta-analyzable subset but also that reporting of clinical descriptors remains incomplete.

The included evidence base was recent but methodologically heterogeneous. Publication years ranged from 2001 to 2026, with a median publication year of 2023; 128 studies were published from 2024 onward. The full-text evidence base included 266 English-language studies and 4 Chinese-language studies. Sample size was extractable for 136 studies. Among those reports, the median sample size was 42 participants (IQR 12-206; range 1-37,740), indicating that many model development studies were small, while a few electronic health record or population-scale studies contributed much larger cohorts.

Study duration, CGM wear duration, recording length, or follow-up time was not reported in a sufficiently standardized way to calculate 1 reliable review-wide duration summary. We therefore extracted duration-related information when available but used forecast horizon, validation design, diabetes type, setting, input modality, and extractable performance uncertainty as the primary harmonized descriptors. This reporting gap is clinically important because a model trained on short-structured CGM segments may not have the same transportability as a model evaluated across longer free-living or inpatient monitoring periods.

The most common prediction targets were short-term future glucose values, hypoglycemia alerts, hyperglycemia alerts, and broader glycemic-excursion outcomes. Reported hypoglycemia thresholds most often corresponded to <70 mg/dL (<3.9 mmol/L), with some studies using clinically important hypoglycemia thresholds such as <54 mg/dL (<3.0 mmol/L). Hyperglycemia thresholds varied more widely, commonly including >180 mg/dL (>10.0 mmol/L) or study-specific higher thresholds. This threshold heterogeneity was one reason threshold-based binary results were treated as supportive rather than definitive.

Model families included recurrent neural networks, long short-term memory networks, gated recurrent units, convolutional recurrent networks, transformer-based models, gradient boosting, random forests, support vector regression or classification, and hybrid or ensemble approaches [1,2,4,29,30,33,34]. Some studies compared machine learning models with naive persistence or conventional forecasting baselines. Others reported only the best-performing model. This variation limited direct model-family comparison.

Input data also varied. Some models used only recent CGM history, whereas others added insulin delivery, meal information, physical activity, demographic variables, laboratory data, heart rate, or inpatient electronic health record variables [2,4,6,7,33]. Studies with richer inputs were not automatically more clinically deployable because those inputs may be unavailable, delayed, or inconsistently recorded in routine care.

Validation reporting was a recurring limitation. A substantial proportion of studies used internal cross-validation, random splits, or same-cohort held-out testing. Fewer studies used temporal validation, external institutional validation, or prospective evaluation. Calibration, decision-curve analysis, and alert-burden measures were rarely reported, even when binary event prediction was the clinical target.

### Continuous Glucose Forecasting

The primary continuous synthesis included 37 RMSE model-horizon effect estimates. The pooled RMSE was 1.18 mmol/L (95% CI 1.01-1.34; Figure 2 [1,2,13-16,29-32,34] and Table 2). The supportive MAE synthesis included 20 effect estimates and yielded a pooled MAE of 0.90 mmol/L (95% CI 0.78-1.02). The number of effect estimates can exceed the number of contributing studies because multiple models, horizons, or validation strata could be extracted from the same report. These results summarize the analyzable subset and should not be interpreted as universal performance benchmarks across all glucose prediction tasks.

**Figure 2.**
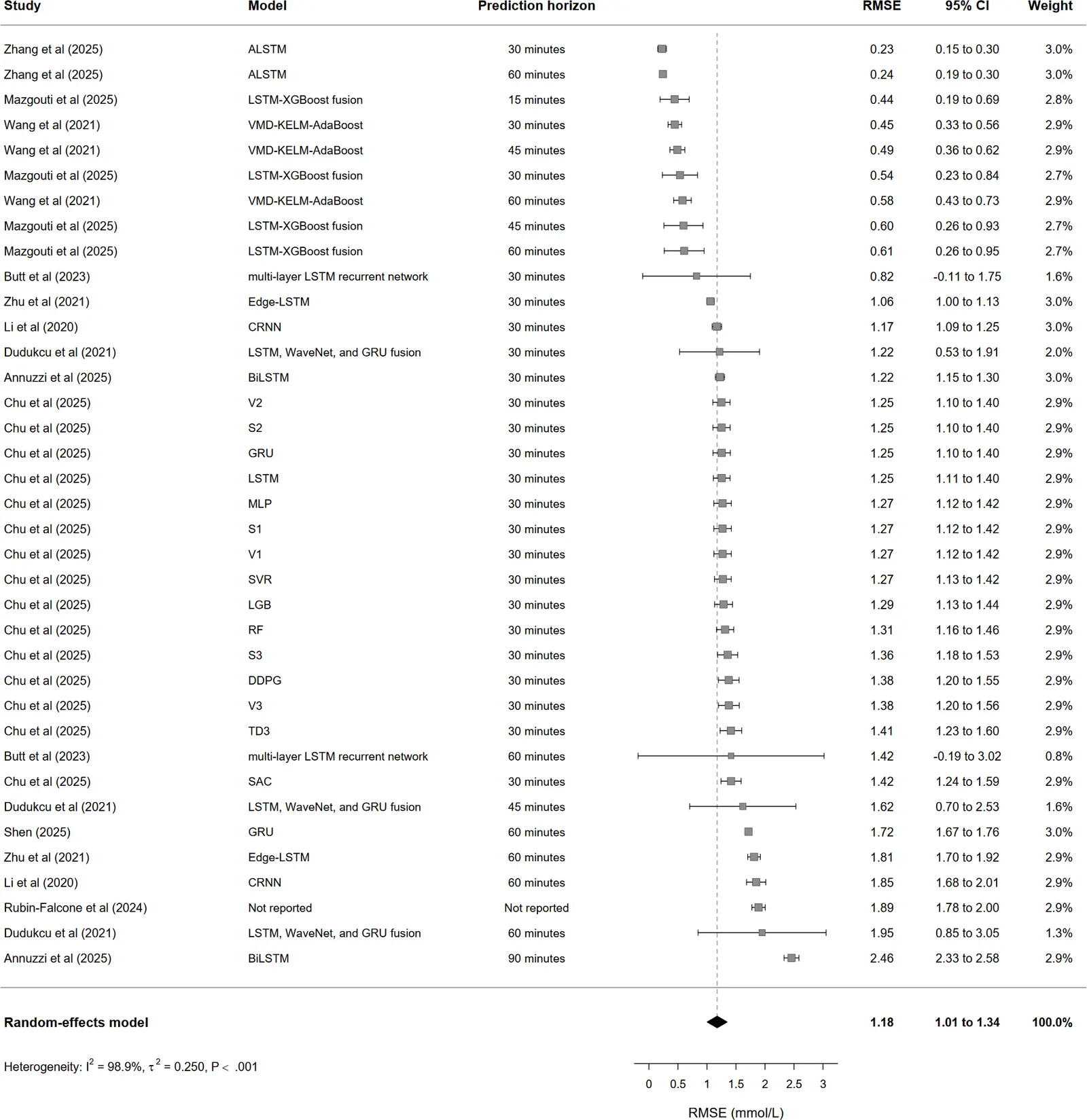
Main random-effects forest plot for RMSE [1,2,13-16,29-32,34]. RMSE: root-mean-square error.

**Table 2.** Main quantitative synthesis results.

Outcome	Role	Effect estimates	Pooled estimate	95% interval^a^	Interpretation
RMSE^b^	Primary continuous	37	1.18 mmol/L	1.01-1.34	Main forecasting summary
MAE^c^	Supportive continuous	20	0.90 mmol/L	0.78-1.02	Supportive error metric
AUROC^d^	Primary binary discrimination	13	0.926	0.877-0.957	Bayesian 95% CrI^e^
Sensitivity	Supportive threshold	10	0.77	0.62-0.88	Direct or reconstructed 2×2
Specificity	Supportive threshold	10	0.80	0.71-0.86	Direct or reconstructed 2×2

a Intervals are 95% CIs, except for the Bayesian AUROC estimate, for which a 95% credible interval is reported.

b RMSE: root-mean-square error.

c MAE: mean absolute error.

d AUROC: area under the receiver operating characteristic curve.

e 95% CrI: 95% credible interval.

Effect estimates are model-horizon or model-threshold analytic estimates. They can exceed the number of contributing studies because several models, horizons, or validation strata may be extracted from 1 source report. The lowest individual RMSE estimates tended to occur in short-horizon or narrowly defined settings, including models evaluated at 15-30 minutes or in structured datasets with dense CGM information [1,30,34]. Higher error estimates were more common at longer horizons or in more heterogeneous settings [4,33]. However, individual ranking should be interpreted cautiously because study design, population, data preprocessing, and validation strategy differed across reports.

Exploratory diabetes-type subgroup analysis separated type 1 diabetes, type 2 diabetes, mixed-population, and unclear-population records (Figure 3A [2,7,13-21,23,25,26,28-47,60] and Table 3). RMSE estimates were lower in the type 2 diabetes subgroup than in the type 1 diabetes and mixed-population subgroups, but the type 2 diabetes estimate was based on fewer source titles and had wider uncertainty. The RMSE subgroup estimates were type 1 diabetes mellitus: 1.18 (95% CI 1.05-1.32; 43 effect estimates, 18 source titles); type 2 diabetes mellitus: 0.89 (95% CI 0.50-1.28; 15 effect estimates, 6 source titles); mixed_both: 1.48 (95% CI 1.26-1.69; 13 effect estimates, 9 source titles); and unclear: 0.61 (95% CI 0.40-0.83; 4 effect estimates, 2 source titles). These findings support separate reporting by diabetes type, but they do not prove intrinsic superiority of models in 1 diabetes type because population setting, treatment context, input modality, and validation design were confounded.

**Figure 3.**
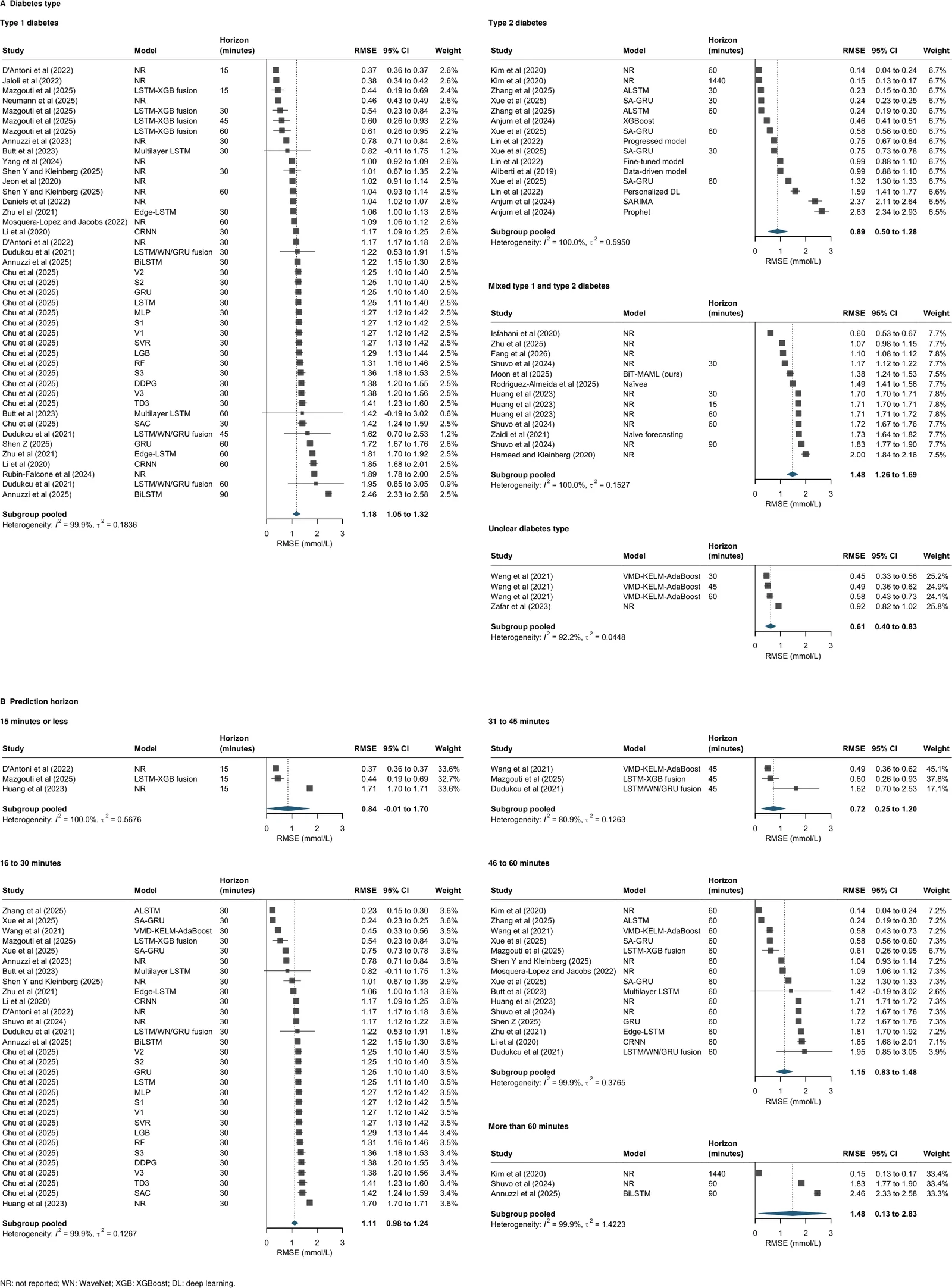
Exploratory RMSE subgroup analyses by diabetes type and prediction horizon. (A) RMSE effects stratified by diabetes type. (B) RMSE effects stratified by prediction horizon in minutes. RMSE values are presented in mmol/L. Squares represent individual model-level effects, horizontal lines represent their 95% CIs, and diamonds represent subgroup-specific random-effects pooled estimates. Subgroup results should be interpreted cautiously because some categories include few source reports or model-level effects. CIs, and diamonds represent subgroup-specific random-effects pooled estimates. Subgroup results should be interpreted cautiously because some categories include few source reports or model-level effects [2,7,13-21,23,25,26,28-47,60]. RMSE: root-mean-square error.

**Table 3.** Exploratory subgroup synthesis results.

Analysis	Subgroup	Effect estimates	Source titles	Pooled estimate (95% CI)
RMSE^a^ by diabetes type	T1DM^b^	43	18	1.18 (1.05 to 1.32) mmol/L
RMSE by diabetes type	T2DM	15	6	0.89 (0.50 to 1.28) mmol/L
RMSE by diabetes type	mixed_both	13	9	1.48 (1.26 to 1.69) mmol/L
RMSE by diabetes type	unclear	4	2	0.61 (0.40 to 0.83) mmol/L
RMSE by horizon	≤15 minutes	3	3	0.84 (−0.01 to 1.70) mmol/L
RMSE by horizon	16‐30 minutes	30	15	1.11 (0.98 to 1.24) mmol/L
RMSE by horizon	31‐45 minutes	3	3	0.72 (0.25 to 1.20) mmol/L
RMSE by horizon	46‐60 minutes	15	14	1.15 (0.83 to 1.48) mmol/L
RMSE by horizon	>60 minutes	3	3	1.48 (0.13 to 2.83) mmol/L
MAE^c^ by diabetes type	T1DM	20	5	0.93 (0.83 to 1.03) mmol/L
MAE by diabetes type	T2DM	16	6	0.93 (0.59 to 1.27) mmol/L
MAE by diabetes type	mixed_both	8	6	1.05 (0.80 to 1.29) mmol/L

a RMSE: root-mean-square error.

b T1DM: type 1 diabetes mellitus.

c MAE: mean absolute error.

Forecast horizon was also analyzed separately (Figure 3B and Table 3). Exploratory pooled RMSE estimates were ≤15: 0.84 (95% CI −0.01 to 1.70; 3 effect estimates, 3 source titles); 16‐30: 1.11 (95% CI 0.98-1.24; 30 effect estimates, 15 source titles); 31‐45: 0.72 (95% CI 0.25-1.20; 3 effect estimates, 3 source titles); 46‐60: 1.15 (95% CI 0.83-1.48; 15 effect estimates, 14 source titles); and >60: 1.48 (95% CI 0.13-2.83; 3 effect estimates, 3 source titles). The 16‐30 minutes and 46-60 minutes categories had the clearest data support; ≤15-minute, 31‐ to 45-minute, and >60-minute groups were sparse. Longer-horizon estimates were therefore interpreted cautiously, and long-horizon forecasting was not pooled with short-term alerting as if the tasks were clinically equivalent.

Input-modality subgroup analysis suggested that CGM-only and CGM-plus-multimodal models had broadly overlapping uncertainty. The exploratory RMSE estimates were CGM_only: 1.20 (95% CI 1.05-1.35; 41 effect estimates, 11 source titles); CGM_multimodal: 1.06 (95% CI 0.83-1.29; 29 effect estimates, 20 source titles); and CGM_insulin_meal: 1.19 (95% CI 0.70-1.69; 5 effect estimates, 4 source titles). Apparent differences should be interpreted as hypothesis-generating because studies differed in population, horizon, and validation design.

MAE subgroup analysis by diabetes type was supportive and less stable than RMSE because fewer main analysis data were available. Exploratory MAE estimates were similar for type 1 and type 2 diabetes, with wider uncertainty in type 2 diabetes. This finding is consistent with the RMSE analysis in showing that type 2 diabetes data exist, but it does not provide enough precision to support a strong claim about diabetes-type differences.

### Binary Glycemic Event Prediction

The principal binary synthesis used AUROC because directly analyzable 2×2 data were limited. The Bayesian pooled AUROC was 0.926 (95% credible interval 0.877-0.957; Figure 4A [8,52-57,60,61]). The posterior distribution of between-study heterogeneity on the logit(AUROC) scale is shown in Figure 4B. A frequentist REML sensitivity model gave a nearly identical point estimate, supporting numerical stability of the AUROC result under the chosen scale and dataset. Figure 4C compares the pooled AUROC estimates from the conservative main-analysis and maximal datasets. Supportive threshold-based meta-analysis yielded pooled sensitivity of 0.77 (95% CI 0.62-0.88) and specificity of 0.80 (95% CI 0.71-0.86; Multimedia Appendix 6). Because these estimates were based on a small number of threshold outcomes and partly reconstructed 2×2 data, they were used for clinical translation rather than as the primary binary benchmark.

**Figure 4.**
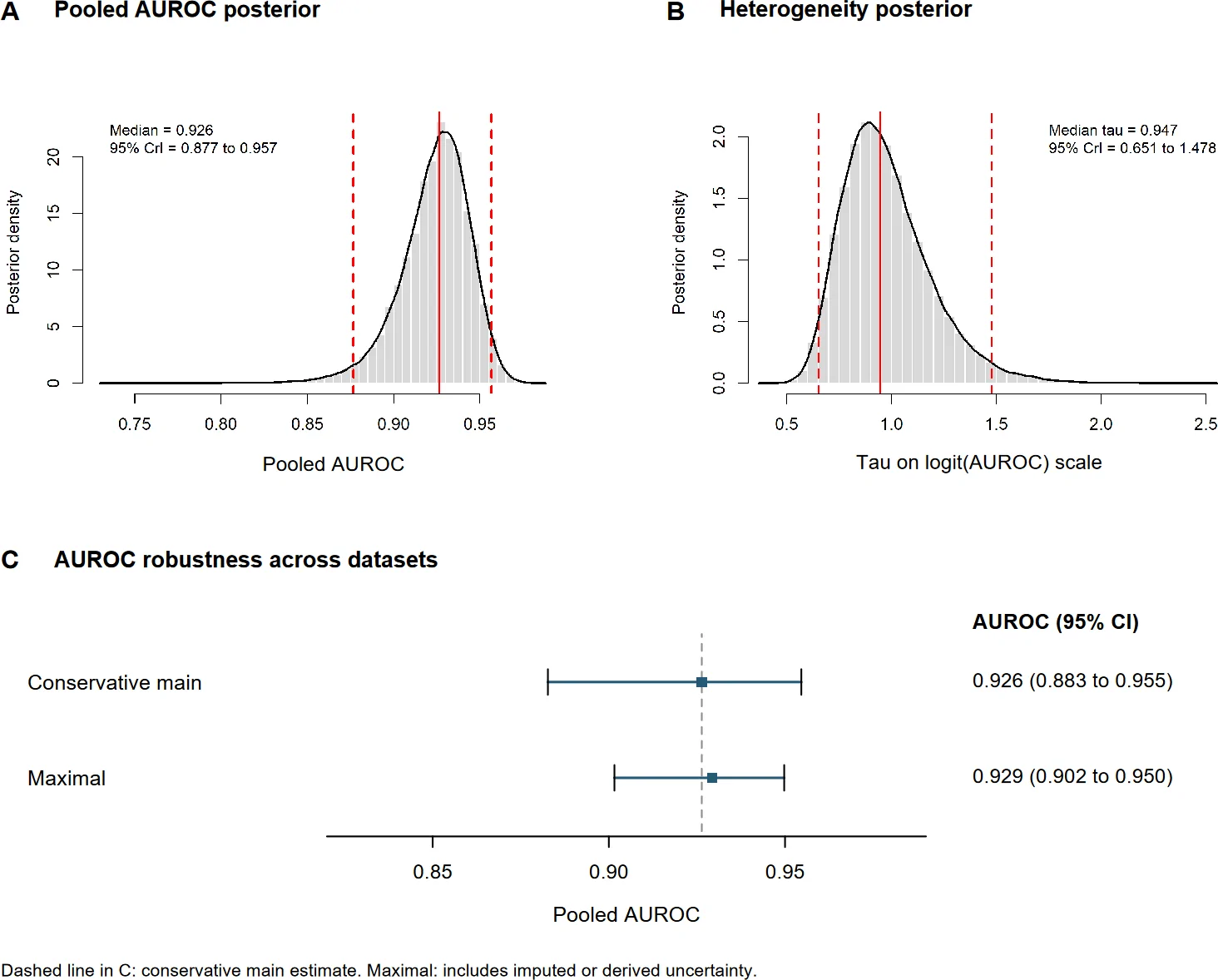
Bayesian AUROC synthesis and AUROC robustness. Panel (A) presents the posterior distribution of pooled AUROC, panel (B) presents the posterior distribution of between-study heterogeneity on the logit(AUROC) scale, and panel (C) compares the conservative main-analysis and maximal datasets [8,52-57,60,61]. AUROC: area under the receiver operating characteristic curve.

The AUROC result indicates that, within the analyzable subset, models could discriminate future event status better than chance. It does not by itself define an alert threshold or prove clinical usefulness. Threshold choice changes sensitivity, specificity, false alert rate, and missed event rate. Therefore, AUROC was paired with supportive sensitivity, specificity, likelihood ratio, and posttest-probability analyses. The threshold-based subset included hypoglycemia and hyperglycemia-related targets with heterogeneous definitions. Some studies used standard biochemical thresholds, whereas others used study-specific adverse-event or high-risk excursion definitions. This heterogeneity limited the feasibility of a single diagnostic-test-style meta-analysis and justified retaining threshold results as supportive evidence.

### Clinical Translation

The pooled positive likelihood ratio was 3.86 (95% CI 2.16-6.50), and the pooled negative likelihood ratio was 0.28 (95% CI 0.14-0.54). In review-defined pretest probability scenarios of 5%, 15%, and 30%, positive predictions increased estimated posttest probability to approximately 17%, 41%, and 62%, respectively, whereas negative predictions reduced estimated posttest probability to approximately 2%, 5%, and 11%, respectively (Figure 5 and Table 4). These scenarios are illustrative rather than prescriptive; actual clinical usefulness depends on event severity, treatment context, alert burden, calibration, and user response.

**Figure 5.**
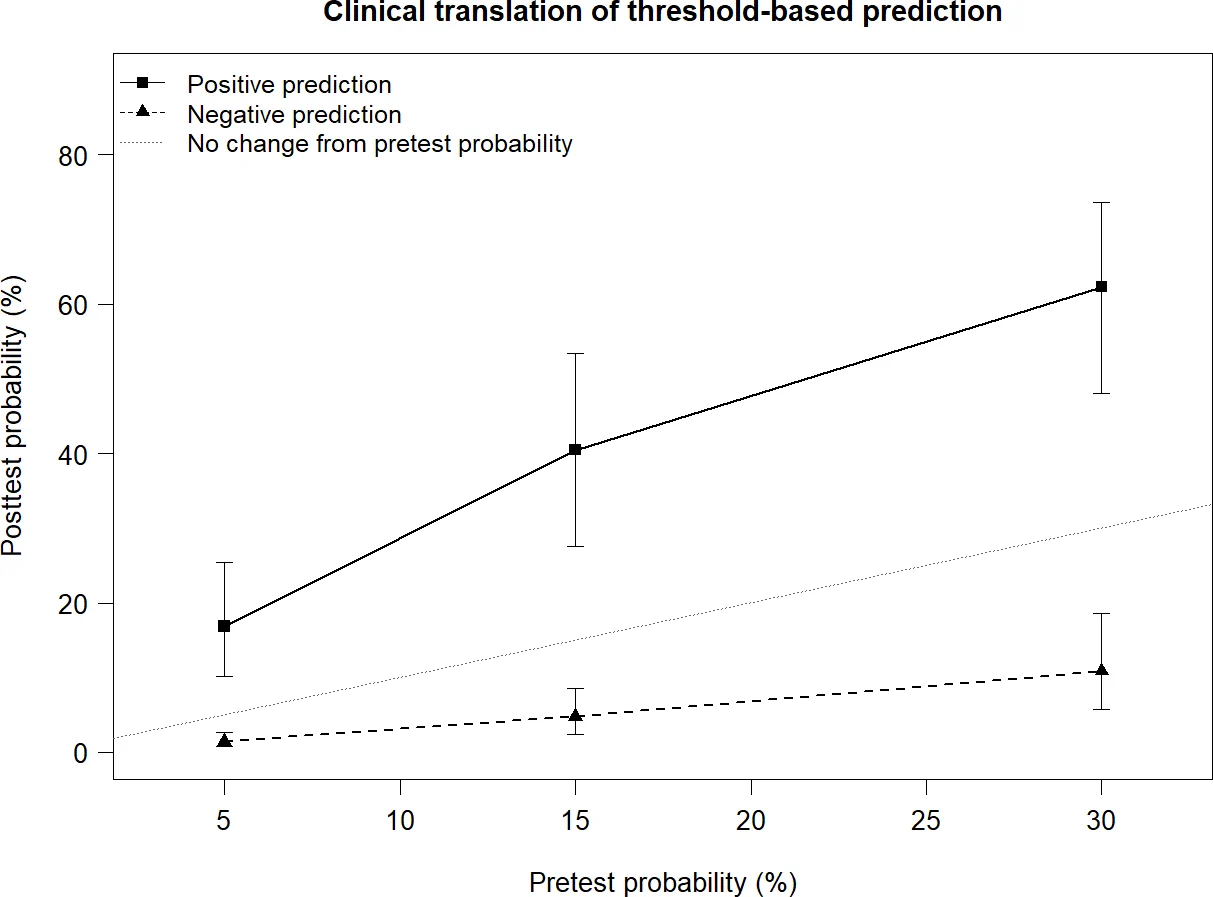
Clinical translation of threshold-based prediction.

**Table 4.** Clinical translation of threshold-based prediction^a^.

Pretest probability	Posttest probability after positive prediction	Posttest probability after negative prediction
5%	16.9% (10.2-25.5)	1.5% (0.7-2.7)
15%	40.5% (27.6-53.4)	4.8% (2.4-8.6)
30%	62.3% (48.0-73.6)	10.9% (5.7-18.7)

a Values within parentheses are approximate uncertainty intervals derived from the limits of the pooled sensitivity and specificity 95% CIs through likelihood-ratio calculations.

These calculations help translate statistical performance into a clinical frame. In a low-risk context, even a positive alert may leave the absolute event probability modest. In a higher-risk context, the same positive alert may be more actionable. Conversely, a negative prediction reduces but does not eliminate risk. This is important for hypoglycemia, where the consequences of missed events may be clinically serious.

The clinical translation analysis should not be interpreted as a treatment recommendation. It assumes that pooled sensitivity and specificity are transportable to the scenario being considered, which may not hold when populations, thresholds, devices, and workflows differ. Prospective validation is needed before these posttest probabilities can be used for clinical decision-making.

### Recent Studies Identified During Evidence Updating

All 6 DOI records in the recent-paper audit were retained in the full systematic review dataset and mapped to diabetes type, setting, and extractable metric availability [3-7,33]. Some provided data usable for descriptive synthesis or expanded exploratory extraction but did not meet the stricter uncertainty or threshold reconstruction requirements for the principal analyses. This distinction is important: absence from a forest plot does not mean absence from the review.

These recent studies strengthened the narrative synthesis by adding examples from type 2 diabetes dietary personalization, hospitalized glucose management, mixed diabetes populations, uncertainty-aware glucose prediction, federated learning, and pooled low-glucose prediction [3-7,33]. Their treatment in the manuscript follows the same rule as all other studies: inclusion in the review depends on topic and population eligibility, whereas inclusion in a forest plot depends on outcome-specific data readiness.

The audit also clarified the difference between search completeness and data usability. A study can be fully eligible for the systematic review and yet remain outside an outcome-specific meta-analysis if it does not report uncertainty-ready RMSE, MAE, AUROC, or reconstructable threshold data.

### Source Fidelity, Dataset Overlap, and Prediction Horizon

The source-fidelity audit preserved 80 effect rows from 19 quantitative-source reports, with a page reference and supporting excerpt for every row. It corrected report-level values where an isolated best-participant result had been carried forward instead of the intended report-level summary, removed invalid use of RMSE as an uncertainty proxy, rejected nonunique exact 2×2 reconstructions, and kept simulation-only evidence outside the primary clinical analysis. The report-level provenance audit identified Dudukcu et al [29], Zhu et al [13], Butt et al [2], Shen [15], and Annuzzi et al [31] as members of the confirmed OhioT1DM dataset family. The locked source membership included Zhang et al [16] (R0490). Seven internally contradictory records from Russon et al [54] and Piersanti et al [53] were retained in the audit trail; contradictory 2×2 records were excluded, whereas directly reported AUROC records remained eligible because the conflicts concerned sample size or sensitivity rather than AUROC.

In the primary within-report analysis, RMSE increased by 8.67 mg/dL for each additional 30 minutes of prediction horizon (95% CI 3.84-13.50; *P*=.006), equivalent to 0.481 mmol/L (95% CI 0.213-0.749). This estimate was based on 12 nonzero contrasts from 8 reports. The direction was not robust to the most conservative overlap handling: clustering by dataset family gave the same point estimate but a 95% CI of −5.64 to 22.99 mg/dL (*P*=.12), and excluding the OhioT1DM family yielded 3.77 mg/dL per 30 minutes (95% CI −3.83 to 11.37; *P*=.21). The continuous result therefore supports a possible horizon-related increase in forecasting error but remains exploratory and sensitive to shared-dataset assumptions (Figure S1A and B and Table S18 in Multimedia Appendix 7 [1,2,7,8,13-16,29-32,34,53-58]).

### Clinical Readiness Evidence Across the Full Review Set

The dual-review audit contained final consensus judgments for all 2160 study criterion records. The 2 reviewers (LF and LL) agreed on 2078 records (96.2%; Cohen κ=0.903). There were 82 initial disagreements, and 160 records required explicit consensus because of disagreement or an Unclear or Not applicable judgment; all were resolved before analysis. Real patient data were identified in 99.2% (258/260) of classifiable studies, participant-level or temporal test separation in 18.7% (44/235), external validation in 4.0% (10/251), prospective evaluation in 0.7% (2/270), calibration assessment in 4.8% (13/270), threshold-specific operating performance in 32.4% (83/256), decision-curve or net-benefit analysis in 2.6% (7/270), and implementation or human-factors evaluation in 2.6% (7/270). Twelve studies met none of the 8 criteria, 138 met 1, 83 met 2, 28 met 3, and 9 met 4; no study met 5 or more. Thus, use of real patient data was common, but evidence of external, prospective, calibration, decision-analytic, and implementation readiness was sparse (Figure S1C and D and Table S19 in Multimedia Appendix 7).

### Risk of Bias and Applicability

The 19 quantitative-source reports yielded 28 PROBAST assessment units after separating analyses that differed by population, validation setting, or model development scenario. Across 560 signaling-question ratings per reviewer, observed agreement was 83.8% (Cohen κ=0.78, 95% CI 0.68-0.85; Gwet AC1=0.80, 95% CI 0.71-0.86). Agreement was lowest for the Analysis domain (74.6%; κ=0.67, 95% CI 0.49-0.80). The reviewers initially differed on 91 signaling-question ratings and 9 domain judgments; all were resolved by consensus after full-text and rule recheck. The domain-specific agreement estimates and CIs are shown in Figure S1D in Multimedia Appendix 8.

After consensus, 24 of 28 units were at High overall risk of bias, 2 were Unclear, and 2 were Low. The distribution was driven mainly by the Analysis domain, which was High in 24 units, Unclear in 2, and Low in 2. Recurrent concerns included inadequate protection against participant- or window-level leakage, unclear separation of tuning from final testing, small effective participant or event samples, incomplete handling of repeated observations or missing data, and absent or incomplete calibration and validation reporting. Overall applicability concern was Low in 18 units, Unclear in 4, and High in 6. Unit-level and summary judgments are presented in Figure S1A-C in Multimedia Appendix 8 [2,8,14,15,29-32,52,53,57,58]. A risk-of-bias–restricted sensitivity analysis was evaluated at the independent-study level rather than treating the 28 assessment units as independent studies. Excluding High-risk units left no eligible non-High independent studies for reliable RMSE or MAE reestimation. Only 2 independent studies remained for AUROC; these results were examined descriptively and were not pooled in an unstable random-effects model.

### Robustness and Reporting Bias

The data-readiness robustness analysis showed similar pooled RMSE estimates across the conservative main dataset, expanded sensitivity dataset, and maximal exploratory dataset: main 1.18 mmol/L (95% CI 1.01-1.34), expanded 1.12 mmol/L (95% CI 0.99-1.24), and maximal 1.14 mmol/L (95% CI 1.02-1.27; Figure S1 in Multimedia Appendix 5). Leave-one-cluster-out analysis did not identify a single study cluster that fully explained the main RMSE finding (Figure S1 in Multimedia Appendix 9).

Funnel plots were produced for outcomes with sufficient effects, but these were interpreted as exploratory because multiple effects could arise from the same study cluster, reporting precision was often derived rather than directly reported, and heterogeneity was high (Multimedia Appendix 10). The funnel plots are therefore used to describe possible small-study or reporting patterns rather than to make strong claims about publication bias. The data-readiness analyses support the general direction of the continuous-outcome findings, but they do not remove the underlying limitations of the evidence. Similar pooled estimates across analysis tiers mean that the main estimate was not driven solely by 1 uncertainty derivation rule; they do not establish low risk of bias or external validity. The data-readiness and risk-of-bias analyses answer different questions and should not be conflated. The former tested whether effect inclusion and uncertainty-derivation rules materially changed the pooled estimate. The latter asked whether a reliable pooled estimate remained after excluding high-risk evidence; for RMSE and MAE it did not, and for AUROC only a descriptive 2-study comparison remained.

## Discussion

### Principal Findings

This review maps 270 studies represented by 299 reports, but only 19 reports supported at least 1 quantitative synthesis. This gap reflects incomplete uncertainty reporting, heterogeneous outcomes and thresholds, dataset reuse, and sparse external validation, limiting the quantitative reuse of an otherwise active literature.

The principal continuous estimate suggests an average RMSE of about 1.18 mmol/L in the analyzable subset. The supportive MAE estimate was about 0.90 mmol/L. These values indicate measurable short-term forecasting performance, but they should not be read as a guaranteed clinical accuracy level. The estimates combine models, populations, horizons, and validation designs that are not interchangeable.

The principal binary AUROC synthesis suggested strong discrimination within the subset that reported usable AUROC uncertainty. However, threshold-based clinical implementation depends not only on discrimination but also on sensitivity, specificity, calibration, event prevalence, alarm burden, and the consequences of missed or false alerts. The posttest probability analysis helps show this dependence in practical terms.

Rather than establishing a universal benchmark, the results distinguish a large model development literature from the smaller body of quantitatively reusable evidence. The PROBAST findings materially qualify these estimates. A high judgment does not mean that every reported model result is false; it means that design, analysis, or reporting features leave an important risk that performance is optimistic or not transportable. Because 24 of 28 quantitative-source assessment units were high, the pooled estimates should be viewed as structured summaries of the available analyzable literature, not as low-bias performance benchmarks for clinical deployment. Internal performance may not transfer across CGM devices, treatment patterns, recording behavior, workflows, or populations; transportability, validation design, and uncertainty therefore matter more than headline performance alone.

### Type 1 and Type 2 Diabetes

Type 1 and type 2 diabetes were not pooled without qualification. Type 2 evidence contributed particularly through recent dietary and inpatient studies [6,7], but fewer type 2 reports were meta-analyzable; its subgroup signal is therefore informative but not definitive. Differences in insulin physiology, treatment, diet, comorbidity, setting, and technology are clinically plausible, but aggregate subgroup comparisons remain observational and confounded. We therefore do not imply that either diabetes type is intrinsically easier to predict. Recent type 2 or mixed-cohort studies broadened a largely type 1 diabetes-centered field [4,6,7,33], but many lacked uncertainty-ready RMSE, MAE, AUROC, or 2×2 data and could not enter formal synthesis. Primary studies should report diabetes type explicitly and provide stratified results for mixed populations when feasible; otherwise, apparently favorable performance remains difficult to interpret clinically.

### Forecast Horizon and Clinical Use

Forecast horizon changes the clinical task: 15- to 30-minute predictions may support immediate alerts, whereas >60-minute predictions require different inputs and assumptions. We therefore reported horizon-specific summaries rather than treating all horizons as equivalent. Short-horizon or narrowly defined settings often appeared more accurate, but this may reflect easier horizons, benchmark reuse, richer inputs, or internal validation. The review cannot identify a universally superior model family; external validation remains more informative for clinical credibility. Long-horizon studies remained in the review but were not used to justify short-term alerting claims, preserving completeness without clinically inappropriate pooling. Short horizons exploit stronger glucose autocorrelation but allow less intervention time; longer horizons offer more time but depend on assumptions about meals, insulin, activity, and physiology. The preferred horizon therefore depends on intended use.

The continuous horizon analysis complements the categorical summaries by controlling for each report’s 30-minute baseline. Its primary slope suggests that forecasting error may rise meaningfully as the prediction window lengthens, but the loss of precision under dataset-family clustering and after removal of OhioT1DM reports shows that the apparent association is partly dependent on a small, overlapping evidence base. The finding should therefore guide study design and reporting rather than be interpreted as a universal conversion rule between forecast horizon and RMSE. The full-review clinical-readiness audit shows that publication volume and use of real patient data have not yet translated into evidence suitable for routine deployment. External validation, prospective evaluation, calibration, decision-curve analysis, and implementation or human-factors assessment were each uncommon. These gaps help explain why favorable discrimination or forecasting error cannot, by itself, establish clinical usage or transportability.

### Clinical Implications

These findings support cautious interest, not benchmark-driven adoption. Alert usefulness depends on baseline risk and false alert burden, and a negative prediction does not eliminate risk or replace symptoms, clinical judgment, or device safety rules [5,7,8]. We found no randomized trials showing improved patient-important outcomes. Translation requires prospective validation, calibration, alert fatigue assessment, and evidence that model-guided action improves outcomes without harm. Error consequences are context-specific: false negatives may delay intervention, whereas false positives may cause alarm fatigue, unnecessary carbohydrate intake, or distrust. Low average RMSE can also conceal clinically important errors during rapid changes or near decision thresholds. Implementation must fit workflow. Outpatient users may need simple alerts; inpatient output interacts with nursing, insulin protocols, nutrition, and electronic records. Transfer across workflows requires validation.

### Reporting and Methodological Implications

The main barrier to stronger synthesis was not a lack of published models but a lack of meta-analysis–ready reporting. Future studies should report diabetes type, treatment context, cohort source, sample size, event threshold, forecast horizon, train-test separation, calibration, uncertainty for all primary metrics, and enough threshold information to reconstruct TP, FP, FN, and TN when binary prediction is central. Dataset reuse should be disclosed explicitly. Public benchmark datasets are valuable, but repeated development and evaluation on overlapping data can inflate apparent certainty if each effect is treated as independent. The study-cluster approach used here is a practical response to that problem, but it cannot fully replace better primary study reporting. Primary studies should also explain how errors such as RMSE and MAE were calculated. At minimum, reports should state the prediction horizon, unit, test set, number of predictions, whether errors were averaged per prediction or per participant, and whether uncertainty was estimated across participants, folds, bootstraps, or independent datasets. Without this information, even familiar metrics become difficult to synthesize.

For binary prediction, authors should report the event threshold, prediction window, decision threshold, TP, FP, FN, TN, sensitivity, specificity, AUROC, calibration, and CIs. Reporting only accuracy or *F*_1_-score is insufficient for clinical interpretation because these metrics depend strongly on event prevalence and class balance. The field needs prospective external validation and shared reporting templates. Authors should share code and preprocessing when possible and identify public dataset versions, participant criteria, train-test splits, and overlap with prior studies.

The major model families have different methodological strengths and limitations (Table 5). Recurrent architectures suit temporal glucose data, whereas tree-based and kernel methods can be practical for structured clinical variables. Transformer and hybrid models can integrate longer-range or multimodal information but require more data and may be harder to interpret. Because populations, targets, horizons, inputs, and validation designs differed, this comparison is descriptive rather than a performance ranking [1,2,4,14-16,29-34,52,53,58].

**Table 5.** Comparative methodological characteristics of major machine learning model families used in glycemic prediction. This table provides a narrative methodological comparison rather than a quantitative ranking. Standardized head-to-head comparisons across model families were not feasible because studies differed in populations, prediction targets, forecast horizons, input modalities, validation designs, and reporting completeness.

Model family	Main strengths	Main limitations	Interpretability approaches	Clinical and reporting considerations
Recurrent neural networks (RNNs^a^, LSTMs^b^, and GRUs^c^)	Model nonlinear temporal dependencies in sequential CGM^d^ data and support short-term or multihorizon forecasting.	Require careful tuning and sufficient data; hidden states are difficult to interpret; and results may depend on window length and data splitting.	Time-step attribution, saliency, integrated gradients, or SHAP^e^-style feature attribution.	Report input-window length, forecast horizon, missing-data handling, train-test separation, and external validation.
Convolutional and convolutional-recurrent networks	Capture local temporal patterns and can combine automated feature extraction with sequence modeling.	Results depend on convolutional window and architecture; local patterns may not capture longer-range physiology.	Saliency maps, temporal activation maps, integrated gradients, or feature attribution.	Report receptive field, preprocessing, sampling frequency, and stability across horizons and devices.
Transformer-based models	Represent longer-range dependencies and heterogeneous or multimodal inputs, including interactions across time.	Often data- and computation-intensive, vulnerable to overfitting in small cohorts, and attention is not a causal explanation.	Attention visualization combined with attribution or perturbation analyses.	Report model size, regularization, calibration, explanation stability, and external or temporal validation.
Tree-based and boosting models (random forests and gradient boosting)	Handle nonlinear relationships, mixed clinical variables, and engineered features; often practical for tabular data.	Raw CGM sequences usually require temporal feature engineering; importance estimates may be biased by correlated predictors.	Global or local feature importance, permutation importance, partial dependence, or SHAP.	Report feature engineering, missing-value treatment, explanation background data, and calibration.
Kernel-based models (SVM^f^ and SVR^g^)	Can perform well in small-to-moderate datasets with engineered high-dimensional features.	Sensitive to scaling, kernel choice, and hyperparameter tuning; probabilities and explanations may be less intuitive.	Coefficient inspection for linear kernels, sensitivity analysis, local surrogates, or SHAP-style methods.	Report scaling, kernel, tuning, class-imbalance handling, and independent test-set performance.
Hybrid and ensemble models	Combine complementary temporal, nonlinear, and multimodal components and may improve robustness across tasks.	Implementation is complex; component contributions and failure modes may be difficult to isolate; and reproducibility can be limited.	Component-wise analysis, ablation studies, ensemble contribution analysis, or model-appropriate attribution.	Report every component, fusion rule, ablation results, computational burden, and code or preprocessing availability.

a RNNs: recurrent neural networks.

b LSTMs: long short-term memories.

c GRUs: gated recurrent units.

d CGM: continuous glucose monitoring.

e SHAP: Shapley additive explanations.

f SVM: support vector machine.

g SVR: support vector regression.

Interpretability should match the architecture and clinical use. Feature importance, permutation methods, SHAP, attention, saliency, and shapelets may identify influential glucose, insulin, meal, activity, or clinical inputs. These model- and data-dependent explanations can be unstable under correlated predictors or distribution shift and are not causal. Studies should report the method, reference data, local or global scope, stability, and clinical evaluation [4,31,58].

### Strengths and Limitations

Strengths of this review include a substantially updated search, explicit recent-paper auditing, separation of binary and continuous prediction tasks, diabetes-type subgroup reporting, horizon-specific analysis, unit harmonization, and a transparent distinction between the review-level evidence base and the quantitative subset. For the quantitative-source reports, PROBAST was completed independently by 2 reviewers (HY and DA) with a prespecified unit-splitting rule, an item-level consensus log, and report-clustered agreement intervals. The supplementary materials provide search strategies, screening decisions, extraction rules, data-readiness classifications, and the complete PROBAST audit trail.

Limitations remain in this review. Many studies lacked uncertainty estimates, calibration, or external validation; some diabetes-type and setting classifications remained unclear. Some uncertainty values were derived from CIs, and threshold analyses partly used reconstructed contingency tables. The search ended April 12, 2026. Subgroups were exploratory and noncausal. Excluding high-risk units left insufficient independent evidence for RMSE or MAE pooling and only 2 independent AUROC studies, so risk-of-bias–restricted quantitative robustness could not be established. The full review set describes field breadth, whereas the 19-report subset represents quantitatively reusable evidence. PROBAST therefore focused on these reports, yielding 28 prespecified assessment units. Their risk-of-bias distribution should not be generalized to all 270 studies or treated as representative of the entire evidence map. Some reports did not permit confident separation of type 1 from type 2 diabetes. Retaining an unclear category reduced forced misclassification but left some subgroup analyses underpowered.

Finally, the review did not evaluate the usability, acceptability, or economic impact of prediction systems. These dimensions are important for digital health implementation, but they were rarely reported in the primary studies and were outside the quantitative scope of this meta-analysis.

The continuous prediction-horizon analysis was post hoc, included only 8 reports and 12 nonzero within-report contrasts, and could not recover the covariance between each 30-minute reference and its longer-horizon estimate. Equal weighting was necessary in the primary model because uncertainty was incompletely reported. Although CR2 inference and multiple overlap sensitivities were used, the small number of independent reports and dataset-family clusters limits precision and precludes causal interpretation of the slope.

The clinical-readiness audit was based on what each report documented and may therefore underestimate procedures that were performed but not reported. Unclear and not applicable judgments were excluded from criterion-specific denominators, while the criteria-met count used only final Yes judgments. The 8-item framework was defined for this review to describe evidence breadth; it is not a validated scale, and neither the criteria count nor interrater agreement establishes clinical effectiveness.

### Conclusions

Machine learning models for glycemic prediction in diabetes show encouraging but not yet clinically definitive performance. The evidence base is broad and now reports type 1 diabetes, type 2 diabetes, horizon, and input-modality patterns transparently. However, only a small subset of the literature is currently suitable for formal synthesis. Pooled estimates should be interpreted as structured summaries of analyzable evidence, not as universal clinical benchmarks. More complete uncertainty reporting, stronger external validation, explicit dataset-provenance disclosure, and clearer separation of prediction tasks are needed before these models can be confidently translated into routine diabetes care. Technically promising forecasting and discrimination signals do not yet establish broad clinical transportability. Future studies should be transparent, externally validated, diabetes-type- and horizon-specific, and clinically interpretable.

## Supplementary material

10.2196/100653 Multimedia Appendix 1 Source-specific search strategies and search dates.

10.2196/100653 Multimedia Appendix 2 Main random-effects meta-analysis of mean absolute error (MAE). Forest plot of MAE estimates in the main analysis dataset. MAE values are presented in mmol/L. Squares represent study- or model-level estimates, horizontal lines represent 95% CIs, and the diamond represents the random-effects pooled estimate.

10.2196/100653 Multimedia Appendix 3 Exploratory mean absolute error (MAE) subgroup analysis by diabetes type. Exploratory subgroup forest plot of MAE stratified by diabetes type. MAE values are presented in mmol/L. Diamonds represent subgroup-specific random-effects pooled estimates. Subgroup results should be interpreted cautiously because some categories included limited numbers of studies or model-level effects.

10.2196/100653 Multimedia Appendix 4 Exploratory root-mean-square error (RMSE) subgroup analysis by input modality. Exploratory subgroup forest plot of RMSE stratified by model input modality, including continuous glucose monitoring (CGM)–only, CGM plus multimodal inputs, and CGM plus insulin and meal information. RMSE values are presented in mmol/L. Diamonds represent subgroup-specific random-effects pooled estimates.

10.2196/100653 Multimedia Appendix 5 Root-mean-square error (RMSE) robustness across analysis tiers. Sensitivity analysis comparing pooled RMSE estimates across the conservative main dataset, expanded sensitivity dataset, and maximal exploratory dataset. Points represent random-effects pooled estimates, horizontal lines represent 95% CIs, and the dashed vertical line indicates the main-analysis pooled estimate.

10.2196/100653 Multimedia Appendix 6 Main random-effects meta-analysis of sensitivity and specificity. Forest plots of threshold-based diagnostic performance. Panel A shows sensitivity, and panel B shows specificity. Squares represent study- or model-level estimates, horizontal lines represent 95% CIs, and diamonds represent random-effects pooled estimates.

10.2196/100653 Multimedia Appendix 7 Additional source-verified analyses. Panel A shows within-report root-mean-square error (RMSE) changes across prediction horizons; panel B summarizes the primary and sensitivity estimates; panel C reports the proportion of studies meeting each clinical-readiness criterion; and panel D shows the descriptive distribution of clinical-readiness evidence breadth.

10.2196/100653 Multimedia Appendix 8 PROBAST (Prediction Model Risk of Bias Assessment Tool) assessment of 28 assessment units derived from 19 quantitative-source reports. Panels A and B present risk-of-bias and applicability judgments, panel C summarizes their distributions, and panel D reports interrater agreement with study-cluster bootstrap CIs.

10.2196/100653 Multimedia Appendix 9 Leave-one-cluster-out sensitivity analysis for root-mean-square error (RMSE). Leave-one-cluster-out analysis evaluating the influence of individual study or model clusters on the pooled RMSE estimate. Each row shows the pooled RMSE after omitting 1 cluster. The dashed vertical line indicates the main-analysis pooled estimate.

10.2196/100653 Multimedia Appendix 10 Exploratory funnel plots for reporting bias assessment. Exploratory funnel plots for root-mean-square error (RMSE), mean absolute error (MAE), and area under the receiver operating characteristic curve (AUROC) outcomes. Panels A and B show RMSE in the main and maximal datasets, panels C and D show MAE in the main and maximal datasets, and panel E shows AUROC on the logit scale. These plots were interpreted cautiously because model-level dependence, heterogeneity, and small numbers of studies in some outcomes may affect visual assessment.

10.2196/100653 Checklist 1 This updated PRISMA 2020 checklist maps each applicable reporting item to its location in the revised manuscript or supplementary materials, reflecting the final study selection process, analyses, risk-of-bias assessment, and data availability information.

## Abbreviations

AC1: agreement coefficient 1
AUROC: area under the receiver operating characteristic curve
CGM: continuous glucose monitoring
FN: false negative
FP: false positive
MAE: mean absolute error
PRISMA: Preferred Reporting Items for Systematic Reviews and Meta-Analyses
PRISMA-DTA: Preferred Reporting Items for Systematic Reviews and Meta-analyses of Diagnostic Test Accuracy Studies
PROBAST: Prediction Model Risk of Bias Assessment Tool
REML: restricted maximum likelihood
RMSE: root-mean-square error
TN: true negative
TP: true positive
TRIPOD: Transparent Reporting of a Multivariable Prediction Model for Individual Prognosis or Diagnosis
